# Three steps to the immortality of cancer cells: senescence, polyploidy and self-renewal

**DOI:** 10.1186/1475-2867-13-92

**Published:** 2013-09-11

**Authors:** Jekaterina Erenpreisa, Mark S Cragg

**Affiliations:** 1 Latvian Biomedical Research & Study Centre, Riga LV-1047, Latvia; 2 Antibody and Vaccine Group, Cancer Sciences Unit, Faculty of Medicine, General Hospital, University of Southampton, Southampton SO16 6YD, UK

**Keywords:** Tumour cells, DNA damage, Senescence, Polyploidy, Self-renewal, Reprogramming, Totipotency, Resistance

## Abstract

Metastatic cancer is rarely cured by current DNA damaging treatments, apparently due to the development of resistance. However, recent data indicates that tumour cells can elicit the opposing processes of senescence and stemness in response to these treatments, the biological significance and molecular regulation of which is currently poorly understood. Although cellular senescence is typically considered a terminal cell fate, it was recently shown to be reversible in a small population of polyploid cancer cells induced after DNA damage. Overcoming genotoxic insults is associated with reversible polyploidy, which itself is associated with the induction of a stemness phenotype, thereby providing a framework linking these separate phenomena. In keeping with this suggestion, senescence and autophagy are clearly intimately involved in the emergence of self-renewal potential in the surviving cells that result from de-polyploidisation. Moreover, subsequent analysis indicates that senescence may paradoxically be actually required to rejuvenate cancer cells after genotoxic treatments. We propose that genotoxic resistance is thereby afforded through a programmed life-cycle-like process which intimately unites senescence, polyploidy and stemness.

## Introduction

Accelerated cellular senescence (often simply termed ‘senescence’) has been enigmatic since its first description. It was initially defined as an irreversible growth arrest induced in proliferating cells by a variety of stress stimuli, the most important being telomere attrition, DNA damage [1] and oncogene activation [2]; the latter two paradoxically representing cancer inhibiting and promoting responses, respectively. The biology of senescence and cancer are clearly closely related, although their inter-relationship remains poorly understood [3,4]. Currently, the complex regulation of these processes is thought to occur at the interface of signalling pathways involved in growth-arrest (p16INK4a/Rb and p19ARF/p53) and promotion (mTOR) [2,5,6].

Phenotypically, the features of accelerated senescence overlap with those of replicative senescence caused by telomere shortening; namely enlarged and flattened cell shape, increased cytoplasmic granularity, polyploidy, and expression of senescence-associated β-galactosidase (SA-β-gal) [7,8]. Hypertrophic senescent cells are also immunomodulatory and secrete cytokines [4]. Perhaps paradoxically, senescent cells can be cleared by CD4+ T cells and macrophages; however, if the immune response is suppressed, cancer develops [9,10]. The question then arises: Why do senescent cells which do not proliferate, pose a cancer risk and require elimination? This raises the possibility that at least a proportion of these cells can revert from terminal senescence [11]. In this article we review the recent evidence supporting this possibility and provide a hypothesis for the molecular and biological basis for how reversion may occur through induced polyploidy and reprogramming for totipotency.

### Escape from genotoxic insults is associated with reversible polyploidy

DNA and spindle damage induce polyploidy in tumour cells, particularly when TP53 function is absent or dysregulated. Although previously the induction of polyploidy was viewed as a reproductive dead end, evidence has now accumulated (reviewed in [12-14]) to indicate that this is not the case. Using various DNA or spindle insults the reversibility of induced polyploidy was shown definitively by direct time-lapse imaging [15-18] and/or isolation of the polyploid fraction with subsequent sub-cloning. These cells can return to mitotic para-diploidy, giving rise to damage escape clones providing clonogenicity in vitro [19-21], and rapid malignant growth in vivo [20,21]. The reversible polyploidy observed in these DNA-damaged tumour cells is however a complex, protracted process successfully giving clonogenic escape to only 10^-4^-10^-6^ of the cells [8,20]. First, polyploidisation occurs in 10-50% of the cells, reaching a peak on day 5 post-damage, with ploidy numbers up to 32n. Extensive cell death (by apoptosis or mitotic catastrophe) ensues leaving only 10-20% of polyploid giant cells alive [19-21], some of which undergo successful de-polyploidisation leading to the establishment of the mitotically cycling survivors from days 7–14 post-damage, while the other survivors slowly senesce [17-20]. Subsequent re-treatment of the cells that recover elicits the same process again [19]. This approximate schedule detailed by us for irradiated Namalwa and HeLa cells [22] is also observed in tumour cell lines of multiple types and species treated with different genotoxic stimuli suggestive of a common underlying biological process with absence of TP53 function [19,21] or equivalent loss of the cell cycle control [20,23] a pre-requisite for its success.

Somatic polyploidy (endopolyploidy) can be reversible and irreversible, differing in several key aspects. Irreversible polyploidy [24-26] occurs through re-replication in the absence of mitosis and can reach very high levels of genome duplication (up to several thousand or more), for example in the salivary glands of Diptera [27] and in the giant cells of the rodent trophoblast [28]. In contrast, endopolyploidy of mammalian hepatocytes and cardiomyocytes occurs through aborted mitoses, is less extensive; and typically does not revert [25,26], although it retains this potential [29,30], while transient polyploid mammalian tumour cells, which typically also do not exceed 32n, can revert to mitosis and initial para-diploidy [31-33]. In tumours, this process is induced by DNA or spindle damage and occurs by aborted mitosis - ‘mitotic slippage’ (reset of tetraploid interphase from aborted metaphase) or by a-cytotomic DNA-bridged bi-polar mitosis starting endopolyploidy from bi-nuclearity and often followed by multi-nucleation [22,23,25,32,34].

Another peculiar feature of the transient polyploidy is that the tumour cells thus by-pass mitotic catastrophe (thereby uncoupling the spindle checkpoint from apoptosis) [35] and enter tetraploidy with unrepaired DNA double strand breaks (DSB). During the ensuing polyploidisation cycles these breaks are repaired by homologous recombination, which is also uncoupled from apoptosis [32]. This behaviour supports the idea that entering polyploidy is part of a tightly programmed process that provides a powerful survival advantage to cells carrying DNA damage and that the whole process has a clear purpose.

It should be noted that cell fusions may also give rise to polyploidy [36] or perhaps the parasexual events represent an intermediate step in the process of reversing tumour cell polyploidy, however their importance and sequence in this process is unclear. Similarly, although the means and consequences of the divisions that the polyploidy tumour cells undergo have been extensively studied and discussed [25,34,37,38], currently the contribution and significance of each (for example bi-polar symmetric and asymmetric, reductional, multi-polar (single and repeated) divisions and segregation of whole genomes [21-23,31,39-41]) for clonogenic survival after DNA damage is unclear. However, two of these may well be of central importance: (1) cell divisions with meiotic features – i.e. those featuring cohesed sister chromatids (segregating diplochromosomes or synapsed homologs), such as observed in 4n-8n cells [40-44] and (2) de-polyploidisation of the high ploidy cells (16n-32n) which is completed by budding of para-diploid daughters, and perhaps represents the final stage in the step-wise survival process [17,31,39,42,45-47] (see below).

Our studies revealed that cyclin B1 and Aurora B kinase overexpressed in endopolyploid tumour cells are important regulators of the transition from the normal mitotic cycle to tetraploidy [22,32]. In line with this conclusion, Marxer et al. reported that tetraploid cancer cells are particularly sensitive to inhibition of Aurora B-kinase [48] and that the underlying mechanism is due to mitotic slippage and subsequent endoreduplication.

### Reversible polyploidy coincides with reversible senescence

Accelerated senescence is also a product of DNA damage in treated tumour cells [1] and recent evidence has indicated that it may be reversible [49-52]. Puig et al. [20] have previously suggested that reversible polyploidy of genotoxic-damage induced tumour cells is associated with reversible senescence of the sa-β-gal-positive cells, a proposal supported by Daniel Wu’s group. The latter showed that escape from accelerated senescence in both a p53-null non small cell lung cancer cell-line (NSCLC) in vitro and in primary tumours is due to overexpression of cdk1 [53] and survivin [54] and that aberrant expression of cdk1 promotes the formation of polyploid senescent cells, which are an important intermediary through which escape preferentially occurs [55]. Cdk1 is a catalytic unit of cyclin B1 regulating entrance into mitosis, while Aurora B-kinase alongside INCENP and survivin regulate correct attachment of spindle microtubules to kinetochores [22,56]. As such, it appears that the illicit transition of cells from the mitotic cycle into polyploidy, induced by DNA damage, paradoxically needs mitosis regulators and can be reversed. The reversal of senescence is apparently also induced by the same damage and depends on a common pathway in a diverse array of tumour cells (human lymphoma, cervical and lung cancer, rat colon cancer, and mouse osteosarcoma). Moreover, this transition programme has an additional dimension, most notably its ability to re-activate signalling pathways associated with meiosis and pluripotency.

### Activation of meiotic genes during reversible polyploidy

Our first observations of reversible polyploidy in irradiated TP53 mutant Burkitt’s lymphoma cells induced by DNA damage lead us to propose an analogy with the evolutionarily-conserved ploidy cycles of unicellular organisms [31]. It was based upon the view that the ploidy cycles (reversible polyploidy) of unicellularians evolved from mitosis to cope with DNA damage and served as the evolutionary precursors of meiosis and sexual life-cycles [57,58]. This hypothesis is supported by the close analogy observed between the signalling pathways responding to exogenous and endogenous DSB introduced by DNA damage and the meiotic nuclease SPO11, respectively, evident in the molecular identity between the mitotic G2M DNA damage checkpoint and the recombination checkpoint of meiotic prophase [59]. As clear confirmation of their homology, sterile SPO11 mutants of C. elegans can be rendered fertile by the application of radiation eliciting DNA DSB [60]. The idea that cancer cells may be exploiting processes similar to these ancient unicellurian ploidy cycles to recover from DNA damage and to support their immortality was gradually developed in a series of articles [31,43,61-63] resulting in the concept of a ‘cancer cell life cycle’ assigning germline properties to the recovering cells. In line with this suggestion, up-regulation of key meiotic genes (MOS, REC8, SGO1, SGO2, DMC1, SPO11, SCYP1,2,3, STAG3) was found and associated with reversible polyploidy in TP53-deficient lymphoma, breast, colon, ovarian, and cervical cancer cell lines after irradiation or spindle damage [21,23,43,61,64]. In addition, ectopic expression of some key meiotic genes was also reported in primary tumours, for example MOS in NSCLC [65], DMC1 in cervical cancer [23], SPO11, REC8, SGO1 and HORMAD1 in melanoma [66]. In particular, activation of Mos kinase in tumour cells after DNA damage was reported as being coincident with overcoming prolonged G2-arrest [61,64] and necessary for the recovery of para-diploid descendants from tetraploid cells formed after spindle damage [21,61,63,64]. Key features of meiotic divisions with the meiotic cohesin REC8 linking sister centromeres, the meiotic recombinase DMC1 colocalising with DSB/γH2AX foci, and the omission of S-phase before mitosis were found in some polyploid lymphoma and HeLa cells induced after irradiation [23,43]. Together this information allows us to suggest that prolonged arrest of damaged tumour cells in G2 and their transition from it through aborted mitoses into polyploidy (with its enhanced capacity for DNA repair) as well as its reversal may be associated with the induced meiosis-like programme. In particular, the aberrant accumulation of Mos-juxta-localised cyclin B1 in endomitotic cells, as well as the upregulation of Aurora B-kinase, both involved in many meiotic processes [59] may be critical for this transition towards polyploidy and in preparing for its reversal. Intriguingly, the same complexes (cdk1/ cyclin B1 and Aurora B-kinase/survivin) are also involved in reversing senescence [53-55].

### Accelerated senescence has overlapping molecular pathways with gametogenesis

Further links between senescence and meiosis can be found in the signalling pathways of two prominent proto-oncogenes, *mos* and *ras*. As reported above, *mos* activation is induced by DNA- or microtubule-damaging agents in TP53-mutated somatic tumour cells of various origins in association with their illicit shift to tetraploidy [21,23,61,63,64]. Mos, also known as MAP kinase kinase kinase, is a key driver of meiosis in the animal kingdom [59,67,68]. In female meiosis, activated Mos causes oocyte maturation – inducing the first meiotic division of the oocytes paused at G2 phase-like prophase (by activating cdk1/cyclin B1), triggering interkinesis with suppression of DNA synthesis, and causing the subsequent arrest at the spindle checkpoint of meiosis II. Here, Mos prevents parthenogenesis in the mature oocytes awaiting fertilisation, through the MEK-pMAPK42-Rsk90 complex and also by acting directly on the meiotic spindle [59,63,67-69]. Mos is downstream of Ras in meiosis and equivalent to Raf in the Ras-MEK-MAPK proliferative pathway. All constitutively active downstream effectors of Mos: MEK, MAPK, and p90Rsk, are also able to induce meiotic maturation when microinjected into oocytes [68]. Given its unique and powerful role in meiosis, it is perhaps not surprising that overexpression of Mos in somatic cells can cause an oocyte phenotype [70,71]. However, Mos, like Ras, is also oncogenic [68,71]. Conversely, the same members of the Ras pathway, including Mos, can cause premature senescence through a MEK-MAPK-dependent p16inka4-pRb arrest of proliferation [7] and activate DNA damage signalling [72]. In fact, strong oncogenic signalling through the constitutively active H-ras^Val12^ mutant is routinely used experimentally as a means to rapidly induce senescence [2]. Intriguingly however Ras is required for meiosis as part of the productive germline programme (reviewed in [70,71]) during the switch from meiosis to mitosis when it activates the cleavage divisions after fertilisation of the mature egg [68,71,73]. The activation of the mature oocyte to initiate post-fertilisation or parthenogenetic cleavage cycles also involves the activity of Akt and PKCα, which can be stimulated by activated Ras and likely mTOR [71,73,74], both central regulators of senescence implicated in cancer [75,76].

Ras can also, directly and equivalently substitute for endogenous Mos in frog oocyte maturation [77]. Moreover, mutant H-ras^Val12^ is nearly 100-times more potent at inducing maturation [77]. It, unlike Mos, does not need stimulation by progesterone and can promote entry into meiotic M phase and cdk1 activation independently of Mos [69]. Clearly then the molecular pathways induced by DNA damage and involved in the illicit transition to tetraploidy and accelerated senescence (which should terminate proliferation), are intrinsically associated with the molecular pathways of gametogenesis and early embryogenesis (which, in contrast, can restore immortality and re-initiate the life-cycle) potentially allowing this switch between them.

### Reversible polyploidy is associated with induction of the ESC-type stemness

Since their description, cancer stem cells have been associated with resistance to genotoxic therapy [78,79]. In addition, a stem-like gene signature has been associated with aggressive tumours of various origins in vivo [80-82], while down-regulation is reported to cause suppression of tumour growth and invasion [83]. Typically resistance to therapy is attributed to the intrinsic properties of stem cells, most notably their enhanced expression of ABC drug efflux pumps, augmented DNA repair capacity and resistance to apoptosis [84], however an alternative possibility of stemness induction in differentiating tumour cells has also been proposed [85]. Our own observations on the induction and reversal of polyploidy favour the latter hypothesis. We established that the key pluripotency and self-renewal cassette (OCT4, NANOG and SOX2) was also induced after DNA and spindle damage in several tumour cell types [45,86].

Importantly, the core stemness gene expression cassette (OCT4, NANOG, SOX2) was observed to be induced in the vast majority of G2 - 4C cells before any completed cell division, precluding the possibility that rare DNA-damage resistant stem cells had been selected. The induction of stemness by DNA damage was further confirmed after separating phenotypically distinct tumour cell populations possessing or lacking stem cell markers from myeloid [87], hepatocellular [88] and breast tumour cell lines [89]. These studies showed that irradiation of differentiated (non-stem cell phenotype) tumour cells caused phenotypic shift to a stem cell-like state (as confirmed by the appropriate markers), with the associated transcriptional profiles, enhanced clonogenicity, growth as 3D-spheres and xenotransplantation activity. Moreover, Lagadec and colleagues [89] convincingly showed that shift of breast cancer cells (including primary clinical material) to the pluripotency state by ionising irradiation occurs principally in the induced polyploid subpopulation. The link between induced tumour cell endopolyploidy and stemness reported by these authors on breast cancer is in accordance with our data on lymphoma and HeLa cells [86] and so supports the existence of a general mechanism.

We showed that this induction is associated with the transition from the mitotic cycle to tetraploidy and is pre-empted by the appearance of nuclear OCT4 foci at promyelocytic (PML) nuclear bodies which further recruit the other members of the core ESC cassette, while treatment with retinoic acid which suppresses the OCT4 promoter leads to dissociation of OCT4 from PML bodies, loss of nuclear localisation and the absence of Nanog [86]. In accordance with this observation, PML protein was reported to be required for activating chromatin remodelling of the *Oct4* promoter in stem cells [90], while Bartova and colleagues showed in ESC that OCT4 becomes recruited to chromatin at sites of DNA damage [91]. Oct-4 was further shown to be critical for the survival/apoptosis-resistance of murine ES cells subjected to stress through interactions with Stat3 and survivin [92]. The question arises then how these pluripotency factors, induced by DNA damage interact with the senescence machinery.

### Senescence meets with stemness at the DNA damage checkpoint

In experiments with normal IMR90 fibroblasts where pluripotency was induced by retroviral transfection of the Yamanaka factors (oct4, sox2, klf4 and c-myc) [93], a concomitant and prevailing emergence of senescence was seen [4,94], due to the upregulation of the cell cycle inhibitors p16^Ink4a^, p15^Ink4b^ and Arf [95]. These observations are interesting because induction of stemness in tumour cells by DNA damage might have mechanisms in common with how normal cells may be artificially re-programmed to become induced pluripotent stem cells (iPSC).

Subsequent research has shown that chromatin relaxation (by histone deacetylase inhibitors), suppression of ROS, inhibition of mTOR, activation of glycolysis and upregulation of autophagy, all improve iPSC reprogramming efficiency. All of these mechanisms which serve to decrease senescence and increase longevity illustrate that accelerated senescence is an antagonist to, and natural barrier for, reprogramming [6,96]. However, this model does not explain why senescent cells, when allowed to remain in the absence of a fully-functional immune system, result in cancer progression [9-11].

We observed that when IMR90 fibroblasts are grown in normal atmospheric oxygen they undergo limited but appreciable polyploidisation at the pre-senescence stage (very low mitotic index) through accumulation at the G2/M DNA damage checkpoint [97]. A small proportion of these cells (4-6%) overcome the barrier to tetraploidy and simultaneously display signs of DNA damage (γH2AX positivity) [72,98], express the senescence markers p16inka4a (CDNK2a) and p21 (CDKN1a), as well as the self-renewal and pluripotency factor NANOG. Thus, a mixed phenotype of accelerated senescence alongside stemness markers appears at the abrogation of the DNA damage checkpoint during the shift to tetraploidy. In fact, this response is also characteristic for stem cells themselves which lack the conventional G1/S checkpoint but retain the checkpoint at G2/M [99] and can access reversible polyploidy through mitotic slippage uncoupled from apoptosis during stress [100].

Moreover, Mantel et al. [100,101] showed that mitotic slippage in stressed ESC is associated with a peculiar sub-phase, where nuclear cyclin B1 remains undegraded. This same unusual enrichment of cyclin B1 was found in polyploid tumour cells induced by irradiation [32,61], in parallel with the Aurora B-kinase enrichment [22]. Furthermore, overexpression of the catalytic subunit of cyclin B1-was also found responsible for the polyploidy-associated reversal of senescence in lung cancer [55]. It is tempting therefore to link the ectopic expression of Mos (which prevents the degradation of cyclin B1 in meiosis) with the by-pass of mitotic catastrophe and slippage into tetraploidy and endomitosis [61,63] as part of the reprogramming process overcoming senescence. Next, we must consider by which means induced polyploidy can favour stemness.

### Polyploidy, stemness and cancer share a glycolytic metabolism

One of the keys to this puzzle was provided by the recent work of Zhang et al. [47]. Using CoCl_2_ to induce hypoxia, Zhang et al. showed that polyploid but not diploid ovarian cancer cells are resistant to hypoxia and accumulate the hypoxia-inducible factor HIF-1α, a key regulator of glycolysis. Individual polyploid tumour cells selected in this way are positive in sphere formation and tumorigenicity assays, and release small clonogenic descendents by asymmetric division and budding. Growth in the presence of hypoxia indicates a reliance of the polyploid giant cells on a more glycolytic energy generation pathway uniting polyploidy with the stemness phenotype as discussed by the authors [47]. An increased glycolytic flux as an outcome of whole-genome duplication was found in yeast [102] and also reported in endopolyploid mouse hepatocytes and human cardiomyocytes [103]. The preferential use of the aerobic glycolytic energy source by tumour cells discovered by Otto Warburg nearly a century ago is now recognized as a key means of metabolic reprogramming [104], while increased glycolysis coupled to increased nucleotide and lipid synthesis is a hallmark uniting tumours of various origins [105]. Zhang et al. also reported that these CoCl_2_-selected polyploid cells of ovarian cancer lacked sa-gal-β-positivity and that their budded descendents acquired a mesenchymal phenotype [47]. The epithelial mesenchymal transition (EMT), which is a key phenotypic link in cancer progression, is known to be associated with glycolysis/HIF-1α -dependent metabolic shift, conferring a powerful growth advantage for tumours in hypoxic conditions. EMT is itself associated with increased stemness – i.e. phenotypic plasticity, sharing properties with cancer stem cells and metastatic cancer [106]. Thus EMT coupled to the shift in metabolism may be interpreted as a key step away from senescence. Interestingly, c-Myc is known to activate glutamine consumption and many of the genes involved in glucose metabolism contributing directly to the Warburg effect [107]. It is furthermore linked with mTOR – a central activator of the Warburg effect in the HIF-1α-mediated glycolysis signaling network [108].

Moreover, c-Myc is long known as a powerful frequently-activated oncogene conferring immortality to cancer cells [71] and perhaps most critically, it is a key reprogramming gene, targeting a large subset of the ESC- module genes, including telomerase [109]. As detailed above, it is also one of the four Yamanaka transcription factors originally described for the generation of iPSC. In addition, c-Myc directly activates DNA replication [110], with its over-expression uncoupling DNA replication from mitosis, thereby favouring polyploidy [111]. Furthermore, there is evidence that c-Myc is involved in polyploidisation of normal mouse hepatocytes; in particular it was shown that c-Myc accelerates hepatic ploidy in transgenic mouse models [112]. c-Myc also up-regulates Aurora B kinase [113] which is implicated in the maintenance of the malignant state and in mitotic slippage [48]; all effects which could contribute to the induction and maintenance of reversible polyploidy. Therefore, the switch to a glycolytic metabolism involving constitutional activation of c-Myc can be suggested as a key molecular event linking reversible polyploidy to stemness, immortality, and likely EMT phenotype of depolyploidised descendants and as a means of shifting from senescence towards cancer progression. It also worth noting that c-Myc accumulates extensively in the cytoplasm of maturing oocytes, before migrating rapidly into the nucleus upon fertilization [114]. This phenomenon may represent the key mechanism of germline immortalisation [71] that be shared with the programmed reversible polyploidy of tumour cells.

### Induced stemness and accelerated senescence emerge in the same polyploid cells

Since the first observations by Roninson over a decade ago [1], it has been known that DNA damaging drug treatments cause cellular senescence with permanent growth arrest of tumour cells. Moreover, as mentioned above, induced polyploidy is regarded as part of this process. At the same time, there are convincing data (initiated by the observations of David Hansemann and Theodor Bovery more than a century ago) suggesting that aneuploidy resulting from the emergence of tetraploidy is intimately involved in cancer initiation and progression [25,34,38,115]. However, it now seems possible that the induction of stemness in the induced tetraploid cells might be the primary driver of this effect, with genome instability and aneuploidy a secondary event.

The question then becomes how does the senescence machinery interact with that regulating stemness in these polyploid cells? Are they friends or enemies? Here we should consider two different phases of the DNA damage response: (1) the early response occurring at the G2 DNA damage checkpoint and the adaptation into tetraploidy, and (2) the later events in the smaller cohort of tumour cells which display higher levels of polyploidy and finally de-polyploidise by budding. In the former induced stemness becomes coupled with senescence by adapting the tetraploidy barrier [97,116] possibly through the activation of meiotic genes as discussed above. In contrast, in the second phase of the response, stemness is apparently progressed and dissociates from senescence [17,45,47].

### Phenotypic bi-potentiality of the tetraploid tumour cells induced by DNA damage

As already mentioned above, the first stage of the DNA damage response is characterised by bi-potentiality, an observation that is supported from parallel studies on oncogene-induced senescence. Sherman et al. [117] showed that immortalised breast epithelial cells transduced by an oncogene do not undergo terminal growth arrest, but instead display a dual state defined as ‘senescence with incomplete growth arrest’ or SWING, whereby ‘senescent’ cells stained for sa-β-gal simultaneously express the proliferation marker Ki67 and occasionally divide [117]. It is interesting to note that Ki-67-positivity was also described as a feature of polyploidising human trophoblasts emerging initially through restitution cycles (mitotic slippage) [118].

‘SWING’ is dependent on TP53, its downstream cell cycle kinase inhibitor, p21cip1 (CDKN1a) and telomerase competency [117]. Our most recent study of a TP53- and telomerase-functional embryonal ovarian carcinoma PA1 [116] is somewhat in accord with the above. Following etoposide treatment we showed a TP53-dependent induction of the self-renewal factor OCT4A alongside G2 arrest and the induction of the senescence regulator p21cip1. As before, expression of both these factors was observed in the cells at the G2/M checkpoint and continued in tetraploid cells. Highly heterogenous levels of OCT4A and p21cip1 were found in these cells, indicating a maintenance of instable bi-potentiality for the two opposing cell fates. Silencing of TP53 lead to premature diversification of these fates, resulting in highly aberrant multicentrosomal divisions and senescence with up-regulated p16(inka4a) and sa-β-gal, and increased DNA damage signaling (chk2). Interestingly, competitive relationships between OCT4 and p21cip1 were also revealed in ESC [119]. There, the p21cip1 promoter was a direct repressional target of OCT4, leading the authors to propose that this function of OCT4 may contribute to the maintenance of ESC proliferation. Another study [120] treating transformed fibroblasts with etoposide revealed that silencing of p21cip1 paradoxically lead to a decrease of Rad51 repair foci and increased apoptosis, while Zheng and colleagues showed that polyploid cells rewire the DNA damage response and repair networks to escape senescence [121]. Collectively these data suggest that the two opposing regulators (Oct4 and p21cip1) initially cooperate to support DNA damage repair, division potential, and protection from aneuploidy. Our findings in embryonal carcinoma underscore that the process following DNA damage occurs in TP53-functional and telomerase competent tumour cells through an intermediate state, which is bi-potential, unstable and perhaps non-determined in respect of individual cell fates. Apparently, this dynamic type of regulation including stochastic elements may be important for the plasticity of the stem cell-like phenotype [122,123].

### Release of rejuvenated descendants is associated with rejection of senescence by autophagy

In the final stages of depolyploidisation, paradiploid progeny derive from the polyploidy “mother” cell. According to the ‘neosis’ hypothesis proposed by Rajaraman and colleagues [12,124] based on live-cell imaging and sub-cloning studies [17], tumour cell immortality was gained through the acquisition of transient stemness during this process. Accordingly, transient stemness is induced during the generation (“birth”) of rejuvenated de-polyploidised descendants from the senescing polyploid “mother” cell through budding. Weihua and colleagues manually isolated individual mouse osteosarcoma polyploid cells from untreated cultures and showed them to be sa-β-gal-positive, but also capable to grow as spheroids, produce normal-sized cells resistant to chemotherapy, and give rise to tumours and lung metastasis in vivo [125]. Observations by Rajaraman’s group were supported and extended by our findings [45] demonstrating that the nuclear markers of stemness that initially appear in the majority of tetraploid cells induced by DNA damage, only persist and accumulate in rare, highly polyploid (16-32n) cells surviving into the second week post-damage (while the other giant cells undergo mitotic catastrophe, apoptosis or irreversible senescence). In these surviving polyploid cells a-cytotomic multipolar radial division occurs followed by differentiation of subnuclei. Some subnuclei continue to accumulate OCT4/NANOG/DMC1 germline markers, while others halt DNA synthesis, lose these markers and undergo selective autophagic sequestration and degradation within the viable polyploid mother cell. Thus, macroautophagy, a programmed process largely responsible for sa-gal-β-positivity [126], becomes involved in sorting and dismantling the degenerated sub-nuclei and facilitates the release of the rejuvenating subnuclei, which then organise their individual cytoskeleton and cytoplasm and bud away [31,39,45], much as described by others [17,42,47]. These latter authors indicated that a stable EMT phenotype is established in the budding tumour cells. Thus, stemness, which appears first as an instable option in the bi-potential tetraploid cells induced by DNA damage, can pass through several endocycles, performing step-wise recombination DNA repair, followed by segregation and sorting of sub-nuclei, before it becomes stabilised and independent from the polyploid “mother”. It should be noted that during each polyploidisation cycle, cell fate decisions are made, with the majority of cells deleted, as can be seen from the progressively decreasing proportion of cells that reach the limit of polyploidy (32n) [19,31,33]. The high DNA damage, step-wise character with negative sorting, and stochastic acquisition of a stem cell-like state [87] may explain why only a negligible proportion of polyploid cells (10^-4^-10^-6^) are able to accomplish it.

### Immunogenicity of polyploidy cells and its disappearance

Tumour cells are almost *de facto* immunogenic, based upon their inherent mutations, and genomic and proteomic dysregulation. However, overcoming immunogenicity has been recognised as a key hallmark of progressive malignancy [127] being countered by numerous immune-evading tumour mechanisms (reviewed in [128]. To date, the best characterised group of tumour associated antigens are the so-called cancer testes associated (CTA) antigens encoded by genes that are normally expressed only in germ, placenta and embryonal cells, but which become ectopically expressed in various tumours [129]. Furthermore, the expression of immunogenic CTA is associated with poor prognosis [129,130]. Some authors have further associated poor prognosis specifically with the mitotic-meiotic transition involving proteins such as REC8, SPO11 and others [66,131].

For these reasons, the immunogenic CTA proteins have been pursued as targets for therapeutic cancer vaccines. Although clinically disappointing, these studies have heralded in an era where the complexity of the immune system and the multifarious tumour-driven modes of immune suppression and evasion have come to be realised.

However, the question remains: why does the immune system not delete these cancer cells? indicating that they may evade immune control or detection in some way. As a recent illustration, patients which show clear immune responses against major CTA antigens in gastric cancer (perhaps indicative of high CTA levels in the tumour) have a poorer survival [132].

As already mentioned, senescent cells can be cleared by CD4+ T cells and macrophages. This process appears important for tumour control as if the immune response is suppressed, cancer develops [9,10]. Recent work shows that tetraploid cells specifically (in a colon cancer cell-line at least) upregulate the immunostimulatory molecule calreticulin on their cell surface [133] and appear to undergo immune-mediated control and destruction [134]. Therefore, tetraploid cancer cells, which are potentially dangerous precursors of invasive aneuploidy [19-21,34,55] can be detected and controlled by the immune system in a similar way to how senescent cells are controlled. How then do we explain the process by which tumour cells cause relapse after treatment?

One possibility comes from findings associated with EMT and autophagy. It is known that autophagy positively regulates the stem-like phenotype of cancer cells [135]. Moreover, cells undergoing EMT were shown to be able to upregulate autophagic mechanisms which serve to impair target recognition and lysis of tumour cells by cytotoxic T lymphocytes (CTL) [136]. Within the reversible polyploidy process outlined above, autophagy is also upregulated during the senescence/stem cell reprogramming phase and during the generation of the final diploid progeny [45,46]. Therefore, as EMT is also likely to occur during this final de-polyploidisation of giant cells [47] it is possible that autophagy serves a similar role here, reducing the immunogenicity of the polyploid cells and their progeny. Perhaps, the removal of the external cytoplasm of the polyploid mother cell in parallel with the sequestration in autophagosomes of the diminuted sub-nuclei described by us previously [31,45,46] serves to reduce the immunoreactivity of the rejuvenated descendents that are released. In this way the polyploid giant cells expressing immunogenic CTA-associated epitopes may also diminish their CTL-reactivity and potentially avoid immune destruction. Therefore descendants of polyploid cells transferring immortality to the next tumour cell generation need both the re-establishment of the germline programme (to up-regulate telomerase and restore self-renewal) and senescence (to allow restructuring and release of the rejuvenated descendents with the aid of autophagy). Rejection of the germline by the senescent deteriorating polyploid mother cell involves the removal of the neo-immunogenic cell surface allowing escape from the immune system. In fact, this process, replicates exactly that which occurs during a life-cycle. However, the significance of the chromatin diminution which is observed, often extruding the whole sub-nuclei, remains obscure. One possibility by way of analogy is with the emission of the polar bodies during oocyte maturation and activation [137]. Another analogy may be found in the evolutionary karyology of heterokaryotic protozoans.

### Chromatin diminution in the life-cycle of Tetrahymena

In the life-cycles of some ciliates, such as *Tetrahymena*, both the vegetative polyploid macronucleus (MA) and germinative micronuclei (MI) originate in the same cell. The MA is degraded by nucleolysis and autophagy and becomes extruded [138] during the conjugation and meiosis of the MI, whilst the removal of the MA prevents this process (cited from [139]). Interestingly, extensive synthesis of the Rad51 recombinase in the MA is a necessary prerequisite for successive meiosis of the MI [140]. A similar collaboration may be required between persisting and further diminuted sub-nuclei in the late stage polyploid tumour cells, in which the diminuted chromatin is also enriched with Rad51 and Rad52 proteins [45]. As commented by Zhang et al. [47] the budding of the EMT descendants from the polyploid mother is reminiscent of the sporogenesis seen in Radiolaria, in which cycling polyploidy is part of the life-cycle [139]. The analogies with protozoan ploidy cycles give support to the view that these cancer cell life cycles recapitulate some features of the earlier evolutionary ploidy cycles, preserved in some extant unicellular protozoans [141].

### DNA damage can reprogramme tumour cells to totipotency

Since the pioneering studies of Mintz and Illmensee [142] it has been known that the genome of cancer cells can prime embryonal development. The molecular events induced in TP53 deficient tumour cells in response to DNA damage indicate a re-activation of a meiosis-like programme, a fundamental mechanism which serves to maintain germline identity and provide the link between the life-cycles. In addition, the core transcription cassette of ESC appears to be evoked. The question then arises how these two pathways are linked through the DNA damage response. Earlier studies revealed that *Oct-4* expression in the germline is regulated independently from epiblast expression by its distal enhancer [143]. Additional studies [144] showed that the OCT4 transcriptional network might be part of the molecular signature of cells from maternal origin from which the inner cell mass and the ESC-associated pluripotency arise. In this way, Oct4 provides the continuity of the totipotency (life) cycle in normal development. On the other hand, observations [86,91,116] indicate that Oct4 is the first of the core ESC cassette genes to respond to DNA damage both in TP53 wild-type stem cells and TP53 mutant somatic tumour cells. Here the function of the different OCT4 isoforms should be also considered [86,145]. Evidently, both the DNA damage responding and the totipotency carrying functions of Oct4 are evolutionarily coupled and involved in the mitotic to meiotic transition of tumour cells. It can therefore be further suggested that Oct4, in its role as both a DNA damage-responder and totipotency regulator, serves as a link between the early meiosis-initiating and later cleavage-like events of de-polyploidisation and budding that give rise to the rejuvenated descendants.

The feasibility of such a process is also seen from the behaviour of ESC themselves: under special cultivation conditions, both female and male cells show gametogenetic potential: i.e. they are capable of undergoing meiosis, oocyte maturation and parthenogenetic development up to the blastocyst stage [146].

The formation of the endoclone by rejuvenated sub-nuclei which acquire individual cytoplasm and initiate mitoses within a single giant cell [31,45] and the potential of these individual cells to form a sphere and induce malignant growth *in vivo*[47,89,125] is entirely in keeping with the embryonal nature of this process. We previously suggested that to achieve this stage, the tumour cells need to undergo about four endocycles thus reaching the ‘developmental totipotency checkpoint’ [33].

## Conclusion

The failure of current cancer treatments to successfully eradicate metastatic disease, likely results from a misunderstanding of the natural history of cancer. Rather than seeing malignancy as a consequence of Darwinian microevolution driven by stochastic mutations, it can be considered as the result of a programmed response illicitly accessed by a few key mutations. Thus the focus of research is transferred from the bewildering multiplicity of mutations to the key transcriptional programme that is accessed and the underlying epigenetics. This programme appears to have been imprinted through evolution to cope with DNA damage and stored in the evolutionary memory of the genome. The mechanisms which gave rise first to reversible polyploidy and then meiosis and sexual life cycles in unicellurians allowing the transition to multicellularity are in some way recapitulated during carcinogenesis. Unfortunately, it appears that the same programme is stimulated in response to genotoxic treatments, leading to disease relapse.

**Figure 1:**
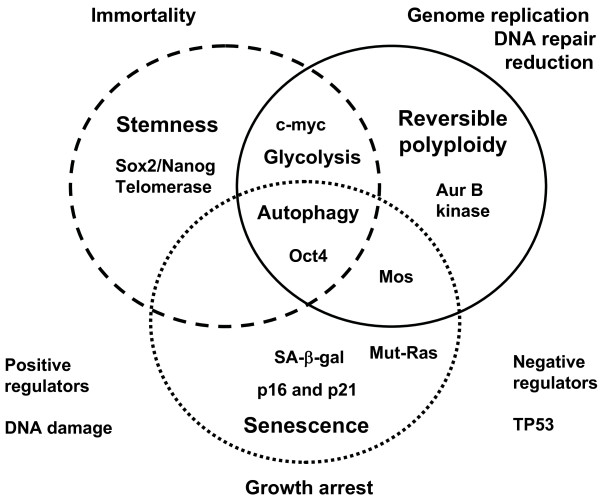
**Inter-relationships between reversible polyploidy, senescence and stemness.** This diagram highlights the inter-relationships and shared molecular pathways between the three processes of polyploidy, senescence and stemness. DNA damage potentiates this process leading to arrest at the G2M damage checkpoint from which cells that by-pass mitotic catastrophe go on to enter the polyploid cycle, eliciting transient stemness to overcome senescence. TP53 serves as a strong negative regulator of the process, favouring arrest at G1, apoptosis induction and inhibiting entry into polyploidy.

The concepts discussed in this review and the latest available data give credence to the existence of an evolutionary ontogenetic relationship between senescence, carcinogenesis and gametogenesis and explain the paradox of involving senescence in carcinogenesis and editing the immunogenicity of tumour cells. This view brings us to a new twist in the centuries-old embryological theory of cancer (reviewed by Erenpreiss [71]) with reversible polyploidy as a new aspect. While trying to unveil the relationships between the overlapping pathways of polyploidy, senescence and stemness (depicted in Figure 1), we have highlighted both the synergism and heterogeneity of opposing regulators, the pleiotropism of key oncogenes and the plasticity of cell fate determination. To fully understand these complex regulations a systems biology approach is required and this has already led to an interesting variant of the embryological theory of cancer where ESC-like state attraction is intrinsically linked to ontogenesis and phylogenesis [147,148]. Recognition that cancer, despite a diverse range of causes and driving mutations, is due to a similar epigenetic acquisition of ilicit transcriptional programmes may favour a shift away from current treatment paradigms to a more holistic whole network approach. This shift is apparently already underway [149-152].

## Competing interests

The authors declare that they have no competing interests.

## Authors’ contribution

Both senior authors have made substantial intellectual contribution to this study. Both authors read and approved the final manuscript.

## Authors’ information

JE and MSC, lead cancer research laboratories in Riga and Southampton, respectively and collaborated both experimentally and theoretically on identifying and understanding the role of reversible polyploidy in cancer resistance over the last 15 years.
